# P07 PCT-targeted ward rounds to reduce inappropriate antibiotic prescribing in medical admissions with suspicion of respiratory tract infections

**DOI:** 10.1093/jacamr/dlac053.007

**Published:** 2022-05-31

**Authors:** Daniel Hearsey, Neil Powell

**Affiliations:** 1 Royal Cornwall Hospital NHS Trust, UK

## Abstract

**Background:**

Lower respiratory tract infections (LRTIs) are a common reason for antibiotics with unnecessary antibiotic prescribing common because of the difficulties differentiating viral, bacterial and non-infectious mimics of LRTIs (e.g. heart failure) due to similar clinical signs and symptoms.^1^ Studies of procalcitonin (PCT)- guided antimicrobial stewardship in managing RTI (including community-acquired pneumonia, hospital-acquired pneumonia, ventilator-associated pneumonia, acute bronchitis, exacerbation of asthma and sepsis with a suspected respiratory tract infection) in various healthcare settings (primary care, emergency departments, hospital wards and ICUs) have shown it to reduce antibiotic exposure and antibiotic associated side effects without impacting treatment failure, hospital length of stay or negatively impacting on mortality.^2^ PCT-guided antibiotic stewardship aims to augment clinical decision-making. We wanted to see whether we could use PCT-guided stewardship to safely stop antibiotics in patients with an RTI diagnosis in those with low severity disease and where bacterial infection looks an unlikely cause for the presenting symptoms.^3^

**Methods:**

An automated daily Excel report sent via e-mail to the antimicrobial pharmacists identified patients with a PCT result and presented the date and levels for PCT, CRP, WBC, any prescribed antibiotics and ward location. Patients with a serum PCT level (<0.25 μg/L) and prescribed respiratory antibiotics were reviewed by one of the antimicrobial pharmacists. All available pathology and radiological results were reviewed on the ward with the patient's clinical signs and symptoms and illness acuity to determine the likelihood of a bacterial cause for symptoms and the risk of early antibiotic cessation. Cases were discussed with the medical team and antibiotics stopped if consensus was achieved. Mortality at 30 days was collected retrospectively for all patients.

**Results:**

Between June 2020 and January 2022, 120 patients with low PCT and on respiratory antibiotics were reviewed. Antibiotics were stopped in 40 patients (33%). Two patients in the early antibiotic cessation group died within 30 days, both of not infectious causes. By comparison, 11 patients in the continuation group died within 30 days.

**Conclusions:**

Pharmacist-delivered PCT ward rounds in patients with a clinical suspicion of respiratory infections successfully reduced inappropriate antimicrobial prescribing without negatively impacting 30 day mortality.
